# Tcf4 Controls Neuronal Migration of the Cerebral Cortex through Regulation of Bmp7

**DOI:** 10.3389/fnmol.2016.00094

**Published:** 2016-10-03

**Authors:** Tianda Chen, Qinwei Wu, Yang Zhang, Tianlan Lu, Weihua Yue, Dai Zhang

**Affiliations:** ^1^Institute of Mental Health, Peking University Sixth Hospital, BeijingChina; ^2^Key Laboratory of Mental Health, Ministry of Health & National Clinical Research Center for Mental Disorders, Peking University, BeijingChina; ^3^Academy for Advanced Interdisciplinary Studies, Peking UniversityBeijing, China; ^4^Peking-Tsinghua Center for Life Sciences, Peking UniversityBeijing, China; ^5^PKU-IDG/McGovern Institute for Brain Research, Peking UniversityBeijing, China

**Keywords:** schizophrenia, brain development, neuronal migration, TCF4, Bmp7

## Abstract

**Background:** Transcription factor 4 (TCF4) is found to be associated with schizophrenia. TCF4 mutations also cause Pitt-Hopkins Syndrome, a neurodevelopmental disorder associated with severe mental retardation. However, the function of TCF4 during brain development remains unclear.

**Results:** Here, we report that *Tcf4* is expressed in the developing cerebral cortex. *In utero* suppression of *Tcf4* arrested neuronal migration, leading to accumulation of ectopic neurons in the intermediate zone. Knockdown of *Tcf4* impaired leading process formation. Furthermore, Bone Morphogenetic Protein 7 (Bmp7) is upregulated in *Tcf4*-deficient neurons. *In vivo* gain of function and rescue experiments demonstrated that Bmp7 is the major downstream effector of *Tcf4* required for neuronal migration.

**Conclusion:** Thus, we have uncovered a new *Tcf4*/*Bmp7*-dependent mechanism underlying neuronal migration, and provide insights into the pathogenesis of neurodevelopmental disorders.

## Introduction

Transcription factor 4 (TCF4) (Gene ID: 6925) is a member of the E-protein family which encodes a highly homologous helix-turn-helix domains. It is expressed in the nervous system as well as the immune system (Einarson and Chao, 1995). Recently, genetic studies have demonstrated that defects in this gene are a cause of Pitt-Hopkins syndrome, a neurodevelopmental disease characterized by mental retardation, seizures, and hyperventilation (Amiel et al., 2007; Brockschmidt et al., 2007). Additional genetic evidence associates TCF4 with schizophrenia-relevant phenotypes (Blake et al., 2010; Li et al., 2010; Lennertz et al., 2011; Quednow et al., 2011; Aberg et al., 2013). TCF4 expression also differs in postmortem brains (Mudge et al., 2008) and blood from schizophrenia patients (Kurian et al., 2011).

Although TCF4 is strongly associated with several neuropsychiatric phenotypes, its role in brain development has not been studied in detail. *Tcf4*(-/-) mice have disrupted pontine nucleus development (Flora et al., 2007). *Tcf4* upregulation enhances the expression of the cyclin-dependent kinase inhibitor gene p57(Kip2) and increases the number of cells in G1 phase among neuronal progenitors (Schmidt-Edelkraut et al., 2014). The major functional studies of TCF4 are in the immune system. TCF4 is essential for the development of B cell and plasmacytoid dendritic cell (PDC) differentiation (Zhuang et al., 1996; Nagasawa et al., 2008).

Here, we report that *Tcf4* is expressed in developing mouse cortex. *Tcf4*-deficient neurons fail to grow leading process, resulting in impaired radial migration. In addition, we show that the expression of *Bmp7* is increased in *Tcf4*-deficient neurons. We further demonstrate that *Tcf4* regulate neuronal migration through *Bmp7*. Overall, our findings establish a new *Tcf4*/*Bmp7* dependent mechanism underlying neuronal migration.

## Results

### *Tcf4* is Expressed in the Developing Cerebral Cortex

**FIGURE 1:**
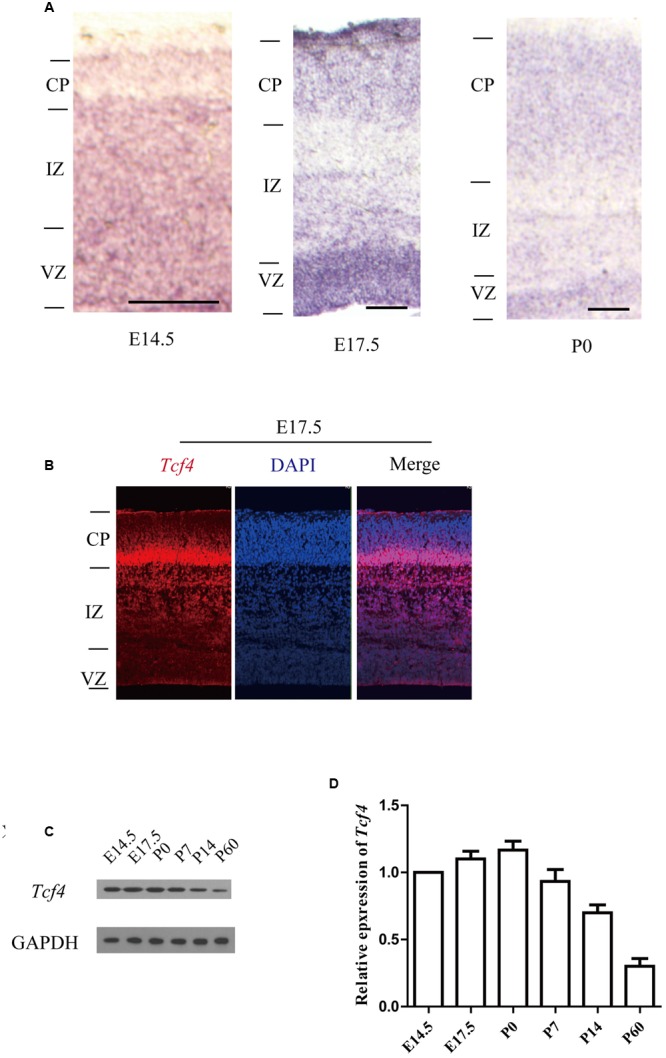
***Tcf4* expression in developing cortical neurons. (A)** *In situ* hybridization shows *Tcf4* expression in the developing cerebral cortex of mice. **(B)** Immunofluorescent staining shows that *Tcf4* is highly expressed in the E17.5 mouse cortex. **(C,D)** Immunoblotting reveals the *Tcf4* protein levels in the cerebral cortex during development (*n* = 3). Scale bars, E14.5 is 100 μm, E17.5 and P0 are 200 μm. Data are shown as the mean ± SEM. one-way ANOVA, followed by an LSD *post hoc* test. CP, cortical plate; VZ, ventricular zone; IZ, intermediate zone.

As a first step in studying whether *Tcf4* plays a role in cortical development, we examined the expression profile of *Tcf4* in the developing mouse brain. Using *in situ* hybridization we found that *Tcf4* mRNA was present in the subplate (SP) and proliferative zones of the cortical wall [the ventricular zone (VZ) and the subventricular zone (SVZ)] at E14 and in the cortical plate (CP) of the cerebral cortex and hippocampus at embryonic (E) day 17.5 and postnatal (P) day 0 (**Figure 1A** and **Supplementary Figure S1**). Immunofluorescent staining also shows that *Tcf4* is highly expressed in the E17.5 mouse cortex (**Figure 1B**). Furthermore, we measured the abundance of *Tcf4* in the developing cerebral cortex and found that high levels of *Tcf4* protein were present in the neocortex from E14.5 to P0 (**Figures 1C,D**).

### *Tcf4* is Essential for Neuronal Migration

**FIGURE 2:**
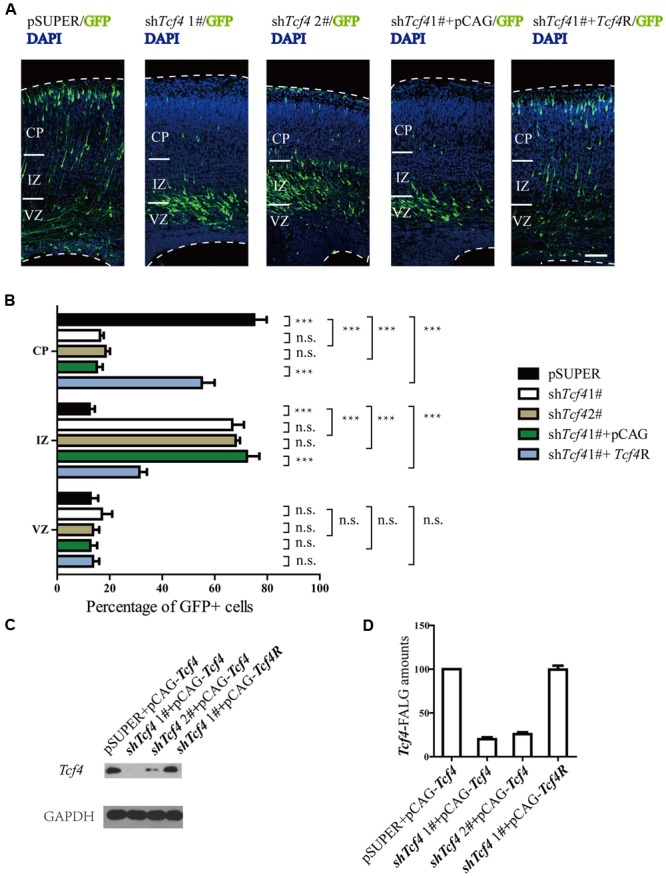
***Tcf4* is essential for neuronal migration in developing cortex. (A,B)** Results of *in utero* electroporation of E14.5 mouse embryos with GFP plasmids together with pSUPER vector (pSUPER) or pSUPER-based shRNAs targeting *Tcf4* (sh*Tcf4* 1# and 2#). Knockdown of *Tcf4* impairs radial migration. Overexpressed *Tcf4*R but not PCAG vector (E14.5-P0) can rescue the radial migration defect caused by *in utero* electroporation with sh*Tcf4* (GFP-positive). Representative coronal brain sections at P0 were stained with antibodies to GFP (green), and counterstained with DAPI, a nuclear marker (blue). The distribution of GFP-positive neurons is quantified (>600 neurons from three mice). **(C,D)** Immunoblot analysis in HEK293T cells shows that *Tcf4-*specific shRNAs, sh*Tcf4* 1# and sh*Tcf4* 2#, are both sufficient to knock down *Tcf4*. Cells were co-transfected with *Tcf4*-FLAG and individual shRNA and total cell lysates were prepared for immunoblotting 48 h after transfection (*n* = 4). Scale bars, 100 μm. Data are shown as the mean ± SEM. n.s., not significant; ^∗^*p* < 0.05, ^∗∗^*p* < 0.01, and ^∗∗∗^*p* < 0.001; one-way ANOVA, followed by an LSD *post hoc* test or two-way ANOVA followed by a Bonferroni *post hoc* test. VZ, ventricular zone; CP, cortical plate; IZ, intermediate zone.

Because *Tcf4* is expressed in the developing cerebral cortex, we wondered whether *Tcf4* may regulate neuronal migration. To test this possibility, *in utero* electroporation was used to cotransfect radial glial progenitors in the cerebral cortex of E14.5 mice with plasmids expressing *Tcf4* shRNA and GFP as a marker. The distribution of GFP-positive cells was examined at P0. Approximately 80% of the *Tcf4* shRNA-expressing neurons were in the IZ and VZ, whereas the control cortical neurons migrated into CP (**Figures 2A,B**). The sh*Tcf4* 1# specificity was demonstrated by the rescue of neuronal migration following co-electroporation of a plasmid-expressing *Tcf4* mutant resistant to the sh*Tcf4* 1# sequences (*Tcf4*^R^) (**Figures 2A–C**). Furthermore, the western blot results showed that sh*Tcf4* 1# caused an 80% reduction of *Tcf4* expression, whereas 2# caused a 75% reduction (**Figures 2C,D**). Thus, *Tcf4* is essential for neuronal migration.

### Knockdown of Tcf4 Impairs Leading Process Formation

**FIGURE 3:**
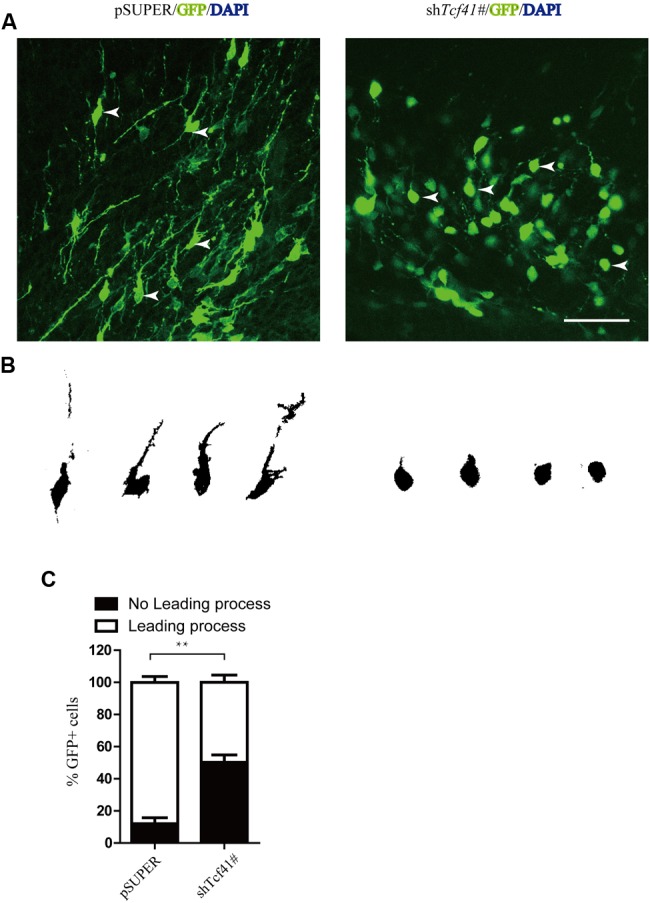
**Knockdown of *Tcf4* impairs leading process formation and neuronal differentiation. (A)** Coronal sections around the IZ from brains electroporated at E14.5. *Tcf4* shRNA inhibits the formation of leading processes at E17.5. Scale bar, 50 mm. **(B)** Tracings of representative transfected neurons from **(A)**. **(C)** Quantification of the experiment shown in **(A)** (*n* = 3). Scale bars, 50 μm. Data are shown as the mean ± SEM. n.s., not significant; ^∗^*p* < 0.05, ^∗∗^*p* < 0.01, and ^∗∗∗^*p* < 0.001; CP, cortical plate; VZ, ventricular zone; IZ, intermediate zone.

To explore the underlying mechanism by which *Tcf4* controls radial migration, we analyzed the morphology of neurons in the IZ in detail. E14.5 mice were transfected with pSUPER and the short hairpin RNA against *Tcf4* (sh*Tcf4* 1#) constructs into the developing mouse brain using *in utero* electroporation. We noticed that *Tcf4* knockdown neurons exhibited impairments in leading processes formation at E17.5 with a large portion of cells (50.3%, compared to 12%in the control) lacking a healthy leading process (**Figures 3A–C**).

We also investigate whether *Tcf4* knockdown might arrest neuronal migration by altering radial glia organization, cell division or cell survival. After 3 days of electroporation, we stained slices with Nestin to indicate radial glia organization, Ki67 to show cell division, and cleaved caspas3 to mark cell survival. There were no significant differences between control and *Tcf4* knockdown slices (**Supplementary Figures S2A–E**). *Tcf4* knockdown thus does not affect radial glia organization, cell division or cell survival.

### *Tcf4* Deficient Neurons Negatively Regulate *Bmp7*

**FIGURE 4:**
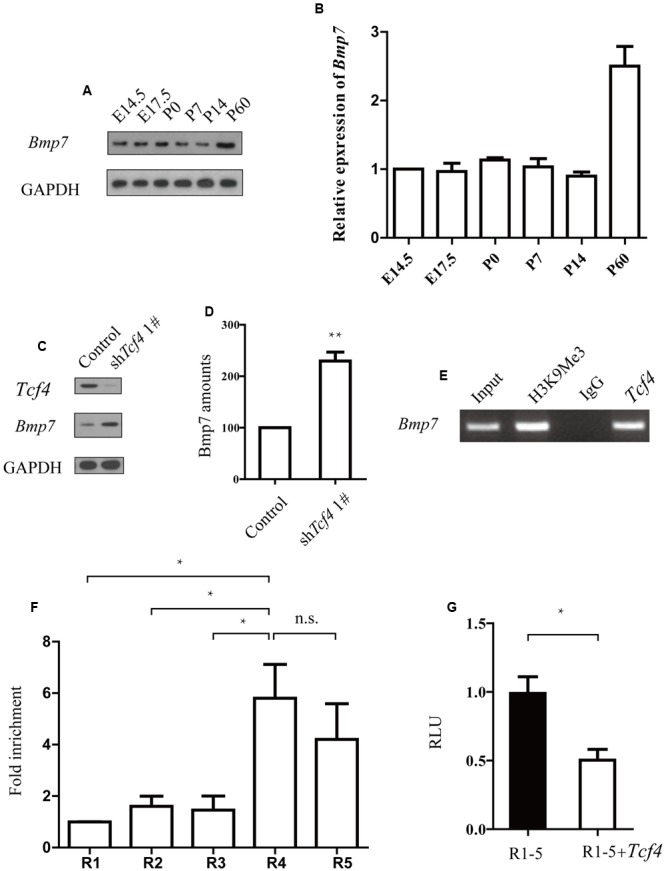
***TCf4* binds to the *Bmp7* promoter and negatively regulates *Bmp7* expression. (A,B)** Immunoblotting reveals *Bmp7* protein levels in the cerebral cortex during development (*n* = 3). **(C,D)** Knockdown of *Tcf4* increased *Bmp7* levels in primary dissociated cortical neurons. Western blot analysis of the protein extracts from dissociated cortical neurons infected with control and *Tcf4*-shRNA lentivirus (*n* = 5). **(E,F)** ChIP analysis using a *Tcf4* antibody and E14.5 cortical tissue detects *Tcf4* binding on -201 bp to +49 bp of the *Bmp7* promoter (R4 and R5) (*n* = 3). (G) Luciferase assays in HEK293 cells transfected with *Tcf4* or a control vector show repression of luciferase activity of the R1–R5 reporter construct by *Tcf4* (*n* = 3) 2 days after transfection. Data are shown as the mean ± SEM. n.s., not significant; ^∗^*p* < 0.05, ^∗∗^*p* < 0.01, and ^∗∗∗^*p* < 0.001; Student’s *t*-test or one-way ANOVA, followed by an LSD *post hoc* test.

Knockdown of human TCF4 is known to affect Bmp7 (Forrest et al., 2013). Here, we found that knockdown of *Tcf4* upregulated *Bmp7* expression in mouse progenitor cells (**Figures 4C,D** and **Supplementary Figure S3**). We also found that the *Bmp7* expression profile in the developing mouse brain negatively correlates with *Tcf4* (**Figures 1B** and **4A,B**). We next identified a putative regulatory region of 1 kb upstream of the transcriptional start site of *Bmp7* which contained six E-box (5′-CANNTG-3′) motifs that are known binding sites of *Tcf4*. Binding of *Tcf4* to these motifs was tested by chromatin immunoprecipitation (ChIP), followed by quantitative real-time PCR using primer pairs (R1–R5) that specifically detected binding to these six motifs (R5 contains two E-box motifs). **Figure 4F** shows that *Tcf4* binds on -201–49 bp of the *Bmp7* promoter (R4 and R5). In addition, we found an enrichment of precipitated DNA of more than 5-fold using a *Tcf4*-specific antibody compared with an immunoglobulin G (IgG) control antibody (**Figure 4E**). Furthermore, this 1 kb regulatory sequence was tested for transcriptional activity by luciferase assays, yielding a repression of 50.5% (**Figure 4G**). This indicates that this sequence conveys functional repression through *Tcf4* binding. Thus, *Tcf4* binds to the *Bmp7* promoter and negatively regulates *Bmp7*.

### *Bmp7* is an Important Downstream Effector of *Tcf4* Required for Neuronal Migration

**FIGURE 5:**
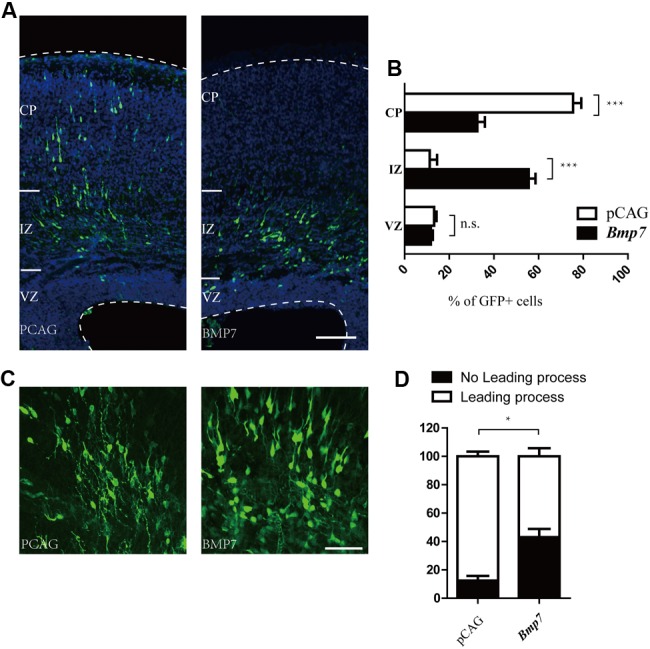
***Bmp7* regulates neuronal migration to the cortical plate. (A,B)** Results of *in utero* electroporation of E14.5 mouse embryos with GFP plasmids together with pCAG vector or pCAG-*Bmp7*. Representative coronal brain sections at E17.5 were stained with antibodies to GFP (green), and counterstained with DAPI, a nuclear marker (blue). The distribution of GFP-positive neurons is quantified (>600 neurons from three mice). Scale bar, 100 μm. **(C)** Coronal sections from brains electroporated at E14.5. *Bmp7* overexpression inhibits the formation of leading processes at E17.5. Scale bar, 50 μm. **(D)** Quantification of the experiment shown in **(C)** (*n* = 3). Data are shown as the mean ± SEM. n.s., not significant; ^∗^*p* < 0.05, ^∗∗^*p* < 0.01, and ^∗∗∗^*p* < 0.001; Student’s *t*-test or two-way ANOVA followed by a Bonferroni *post hoc* test. CP, cortical plate; VZ, ventricular zone; IZ, intermediate zone.

**FIGURE 6:**
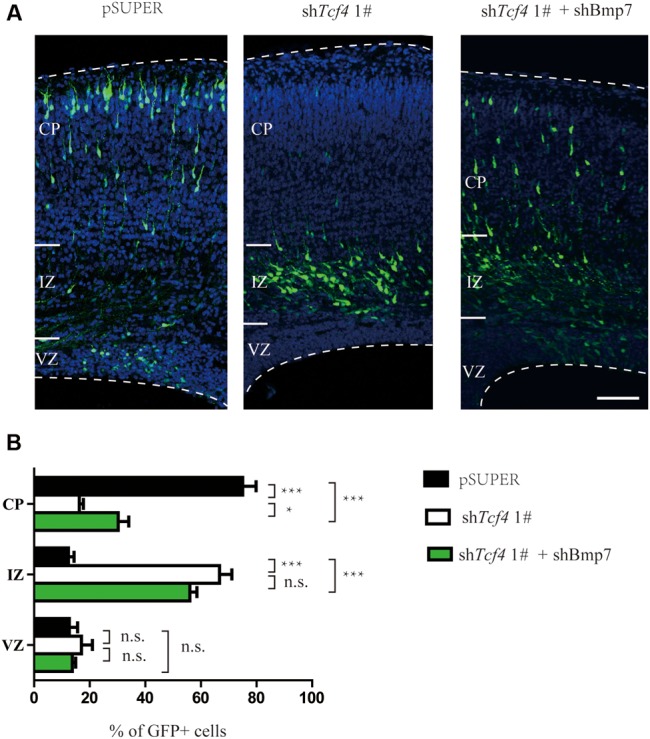
***Bmp7* is an important downstream effector of *Tcf4* required for neuronal migration. (A,B)** *Bmp7* shRNA can partially rescue the radial migration defect caused by *in utero* electroporation (E14.5-P0) with sh*Tcf4* (GFP-positive). The number of GFP-positive neurons in each cortical zone is quantified (>600 neurons in three mice). Scale bar, 100 μm. Data are shown as the mean ± SEM. n.s., not significant; ^∗^*p* < 0.05, ^∗∗^*p* < 0.01, and ^∗∗∗^*p* < 0.001; two-way ANOVA followed by a Bonferroni *post hoc* test. CP, cortical plate; VZ, ventricular zone; IZ, intermediate zone.

Electroporation of *Bmp7* cDNA at E14.5 resulted in migration defects of cortical neurons similar to those observed in *Tcf4*-depleted neurons at E17.5 (**Figures 5A,B**). In addition, the proportion of neurons without leading processes was significantly increased upon overexpression of *Bmp7* in comparison to a control vector in IZ (**Figures 5C,D**). Finally, we performed rescue experiments by introducing *Bmp7* shRNA constructs into *Tcf4*-deficient cells. **Figures 6A,B** show that knockdown of *Bmp7* partially rescued neuronal migration defects in *Tcf4*-deficient neurons. Altogether, our data provide evidence that *Bmp7* is an important downstream effector of *Tcf4*, regulating radial migration of cortical neurons.

## Discussion

Defects in neuronal cell migration in the cerebral cortex can lead to mental retardation, schizophrenia, and epilepsy (Kerjan and Gleeson, 2007; Walsh and Engle, 2010). The human TCF4 gene is implicated in susceptibility to schizophrenia and TCF4 haploinsufficiency is the cause of the Pitt-Hopkins mental retardation syndrome. However, the *in vivo* role of TCF4 in cortical development has remained unclear. Emerging evidence suggests that abnormalities in neuronal positioning are among the underlying causes contributing to the clinical symptoms of these diseases (Moffat et al., 2015). In support of this idea, we show that knockdown of *Tcf4* impairs neuronal migration. The current demonstration opens a new avenue for research on the function of *Tcf4*.

Our data demonstrate that knockdown of *Tcf4* impairs leading process growth. The precise mechanism of leading process is still unknown. The formation of proximal cytoplasmic dilation in the leading process (PCDLP) of migratory neocortical neurons is crucial for somal translocation and neuronal migration (Yang et al., 2012). Interestingly, a large portion of *Tcf4* knock down cells lack the characteristic proximal cytoplasmic dilation presented in most of control neurons (**Figure 3A**). These data suggest that the loss of *Tcf4* may affect PCDLP and thus impair leading process growth and neuronal migration.

Our data do not exclude the possibility that *Tcf4* knockdown may affect other processes of neuronal migration, such as neuronal progenitor cell differentiation (Fischer et al., 2014). It is reported that *Tcf4* interacts with Math1 to regulate differentiation of a specific subset of neuronal progenitors (Flora et al., 2007). Compatible with this, our data demonstrate that knockdown of *Tcf4* impairs progenitor cell differentiation (**Supplementary Figure S4**). However, at E17.5, most of the *Tcf4* depleted neurons leave VZ/SVZ (**Supplementary Figure S5**). Two days later, these neurons still had not migrated into the CP zone (data not shown). These data indicate that, at least for the neurons which have left VZ/SVZ, differentiation deficiency is not the reason why they cannot migrate into the CP zone.

Here, we show that *Tcf4* knockdown upregulates *Bmp7*. We found that *Tcf4* negatively regulates *Bmp7*, which conflicts with a previous report (Forrest et al., 2013). The reason might be the differences between 293T cells and mouse neurons (Segklia et al., 2012; Forrest et al., 2013). Our finding is consistent with previous research showing that BMP7 affects radial neuronal migration (Ortega and Alcantara, 2010). However, this does not exclude the possibility that additional downstream targets of *TCF4* are involved in this process. It has been reported previously that knockdown of TCF4 affects multiple signaling pathways including NOTCH1 and NEUROG2 (Forrest et al., 2013). NOTCH1 has been shown to interact with Reelin signaling and regulate neuronal migration in the cerebral cortex (Hashimoto-Torii et al., 2008). Neurog2 has been found to control two waves of neuronal differentiation in the piriform cortex (Dixit et al., 2014). Thus, it remains to be addressed whether TCF4 transduces other signaling networks to regulate migratory behavior of neurons.

## Materials and Methods

### Plasmid Constructions

The shRNA target sequence of shTcf41# was 5′-GAACGGAGGATGGCCAATAAT-3′. shTcf42# was GGTCAAGATCTAGCAATAACG. α2-shRNA-2 was 5′-ACATATGCCAGTCCTGAAA-3′. Bmp7 was 5′-TCCATCTCCGTAGTATCCG-3′. shRNA sequences were designed and subcloned into pSUPER plasmid. The cDNA of mouse Tcf4 and shRNA-resistant constructs of mutants of Tcf4 were generated with the QuikChange mutagenesis kit (Stratagene) were cloned into pCAG-IRES-EGFP plasmid.

### Cell Culture

For neurosphere cultures, single dissociated cortical progenitor cells (E12.5) were cultured in serum-free DMEM medium with 20 ng/mL FGF2 and EGF (Invitrogen) for 7 DIV. To subclone, neurospheres were collected and gently dissociated using Accutase (Gibco) for 20 min at 37°C. Cells were replated at 100 cells per ul for each condition. The differentiation media was made up of low glucose DMEM with penicillin-streptomycin-glutamine, 2% B27 supplement, and 1% fetal bovine serum (Invitrogen).

### Real-Time PCR

Total mRNAs of the neocortex of E14.5, E17.5, P0, P7, P14, P28, and P60 mice were extracted with TRIzol reagent(Invitrogen). Super-Script II reverse transcriptase (Invitrogen) was used for reverse transcription to produce complementary DNAs (cDNAs). Real-time PCR was performed with the KAPA SYBR FAST qPCR Kits (Kapa Biosystems) and on a Roche LC96 apparatus. Primer pairs are, forward, 5′-TCTTCTCTCAGCCAACAGACAC-3′ and reverse, 5′-TTCAAGTCAGGGGAAGTTGC-3′.

### *In situ* Hybridization

*In situ* hybridization on the brain sections was performed with digoxigenin-labeled antisense riboprobes. Full length cDNA of Tcf4 was amplified with PCR primers and cloned into pGEM-T vector (Promega) to generate antisense and sense probes for Tcf4. The digoxigenin-labeled antisense and sense riboprobes were synthesized by *in vitro* transcription with DIG RNA Labeling Mix (Roche). Mice were perfused with 4% paraformaldehyde (PFA) and fixed overnight in 4% PFA at 4°C. Fixed brains were cryoprotected overnight in 30% sucrose/ phosphate buffered saline (PBS) at 4°C and mounted in OCT compound and sectioned coronally (20 mm) with a cryostat (Leica). *In situ* hybridization was performed as described previously (Wu et al., 2012). Briefly, brain sections were hybridized for 18 h at 65°C. The hybridization signal was detected with an alkaline phosphatase-coupled antibody (1:2,000) against digoxigenin, as well as nitro blue tetrazolium and 5-bromo-4-chloro-3-indolyl phosphate as color reaction substrates.

### Immunostaining

For immunohistochemistry, brain sections were washed in PBS for three times, and blocked in PBS supplemented with 5% BSA and 0.1% Triton X-100 for 30 min at room temperature. Thereafter, the brain sections were incubated with primary antibodies against Cux1 (1:50, Santa Cruz), Ki67 (1:1,000, Invitrogen), Cleaved Caspase-3 (1:1,000, Cell Signaling Technology), Nestin (1:200, Sigma), Pax6(1:100, Millipore), and GFP(1:1000, Molecular Probes), overnight at 4°C. After washing, sections were incubated with the correspondent secondary antibodies for 2 h at room temperature and then counterstained with DAPI (1:2,000, Sigma) for 15 min at room temperature before coverslipping.

### Immunoblotting

Tissues dissected from the mouse brains and cultured cells were lysed in RIPA buffer [20 mM Tris-HCl (pH = 7.5), 150 mM NaCl, 1 mM EDTA, 1 mM EGTA, 1% NP-40, 1% sodium deoxycholate, 1 mM PMSF, 10 mg/ml aprotinin, 1 mg/ml pepstatin A and 1 mg/ml leupeptin]. The equivalent denatured samples were subjected to SDS-PAGE, transferred and probed with antibodies against Tcf4 (1:1,000, proteintech), GAPDH (1:4,000, Abcam). Bmp7 (1:1000, Proteintech), Smad1 (1:1000, Proteintech), phosphorylated Smad1 (1:1000, Abcam), Neurogenin2 (1:1000, Abcam), and FLAG (1:1000, sigma).

### *In utero* Electroporation

E14.5 ICR-strain mice were used for *in utero* electroporation as described previously (Ip et al., 2012). Briefly, E14.5 pregnant mice were anesthetized with 0.7% pentobarbital sodium. The midline laparotomy was performed after the cleaning of the abdomen and the uterus were taken out. DNA plasmids (1–2 μl) of high concentration with 0.05% Fast Green (Sigma) were injected into the lateral ventricle through a polished micropipette. pSUPER shRNA was mixed with pCAG-IRES vector expressing GFP at 1:1 ratio (1 μg:1 μg) with a final concentration of 2 μg/μl. In rescue experiments, pSUPER shRNA was mixed with indicated pCAG-IRES expressing constructs at a 1:2 ratio (1 μg:2 μg) with a final concentration of 3 μg/μl. Square electric pulses were delivered at a rate of one pulse per second to embryos through the uterus by holding them with forceps-type electrodes, while the uterus was kept wet by dropping saline (prewarmed at 37°C) between the electrodes. Five electrical pulses (33V, 50 ms, 1s interval for mice) were applied across the uterine wall using electroporator (ECM-830 BTX). The uterine horns were then replaced in the abdominal cavity and the abdomen wall and skin were sutured using surgical needle and thread.

### CHIP Assay

ChIPs were performed as previously described (Chen et al., 2012), using anti-H3K9Me3 (Abcam), anti-TCF4 (Abcam), anti-IgG (invitrogen), DNA released from the precipitated complexes was amplified by PCR using sequence-specific primers. Primer pairs used in **Figure 5C** are, Bmp7 forward GATCGGAAAGGGGTTTGTTG, reverse ACCCGAGGTCACTTGCTG; β-actin forward, AAATGCTGCACTGTGCGGCGAA, reverse TGCTCGCGGGCGGACGCGGTCTCGG.

### Luciferase Assay

The 1 kb region in the promoter of Bmp7 was cloned into the PGL-3 basic Vector (Promega). This construct was transfected into HEK293 cells with and without pCAG-Tcf4 using Lipofectamine 2000 in accordance with the manufacturer’s instructions (Invitrogen). Renilla was co-transfected in each well as a transfection control. Supernatant from transfected cells was analyzed 48 h after transfection. Luciferase assays were performed using the Dual Luminesence Reporter Assay system (Promega) in accordance with the manufacturer’s instructions and BioTek’s Synergy H1. The values are reported as the mean ratio of luminescence intensity (RLU) of firefly over Renilla. Values were collected from three independent experiments performed with at least three replicates perexperiment.

### Statistical Analysis

All date are presented as the mean ± SEM. Statistical significance was calculated using an unpaired Student’s *t*-test, one-way ANOVA, or two-way ANOVA. Differences were considered significant at *p* < 0.05. Quantification of neuronal migration was estimated by recording GFP positive neurons in distinct regions of the cerebral cortices (CP, IZ, and VZ). Two-way ANOVA followed by a Bonferroni *post hoc* test was used. More than 600 neurons GFP+ neurons from three brains were analyzed in each group. Quantification of morphology of neurons was estimated by recording percentage GFP posictive of neurons with or without leading process. Student’s *t*-test. More than 100 GFP+ neurons from three brains were examined in each group.

### Ethics Statement

All methods were carried out in accordance with the approved guidelines.

All protocols was approved by the Animal Care and Use Committee of Peking University Health Science Center.

## Author Contributions

TC and QW wrote the main manuscript text and prepared all figures. YZ, TL, and WY prepared plasmids. DZ supervised the work. All authors reviewed the manuscript.

## Conflict of Interest Statement

The authors declare that the research was conducted in the absence of any commercial or financial relationships that could be construed as a potential conflict of interest.

## Supplementary Material

FIGURE S1 **(A–C)** *In situ* hybridization shows Tcf4 expression in the developing cerebral cortex of mice. Scale bars, 200 μm.

FIGURE S2 **(A–E)** *In utero* electroporation at E14.5 with sh*Tcf4* or pSUPER as indicated. The results were quantified by counting the number of stained cells in a constant area of each section, and averaged across sections from at least three different embryos for each antibody. Immunostaining at E17.5 for Nestin, cleaved Caspase 3 or Ki67.Scale bars, 100 μm. Data are shown as the mean ± SEM. n.s., not significant; **p* < 0.05, ***p* < 0.01, and ****p* < 0.001; CP, cortical plate; VZ, ventricular zone; IZ, intermediate zone.

FIGURE S3 **(A)** Immunoblot analysis in HEK293T cells shows that *Bmp7-*specific shRNA, sh*Bmp7* is sufficient to knockdown *Bmp7*. Cells were co-transfected with *Bmp7*-FLAG and individual shRNA and total cell lysates were prepared for immunoblotting 48 h after transfection. **(B)** Tcf4 knockdown increased Bmp7 protein levels in primary neuronal progenitor cells. Western blot analysis of the protein extracts from progenitor cells infected with control and Tcf4-shRNA lentivirus.

FIGURE S4 **(A,B)** Immunocytochemistry for Nestin (red) on control and sh*Tcf4* neurosphere cultures after 5 days under differentiation conditions. Scale bars, 100 μm. **(B)** Quantification of Nestin positive cells in **(A)**. Data are shown as the mean ± SEM. n.s., not significant; **p* < 0.05, ***p* < 0.01, and ****p* < 0.001; Student’s *t*-test or one-way ANOVA, followed by an LSD *post hoc* test.

FIGURE S5 **(A,B)** *In utero* electroporation at E14.5 with sh*Tcf4* as indicated. Immunostaining at E17.5 for Pax6 or Tbr2 (markers for VZ). VZ, ventricular zone.
