# Supplementary figures and images for: Tcf4 Controls Neuronal Migration of the Cerebral Cortex through Regulation of Bmp7
Source: Front Mol Neurosci. 2016 Oct 3;9:94. doi: 10.3389/fnmol.2016.00094 (PMC5046712; doi:10.3389/fnmol.2016.00094)

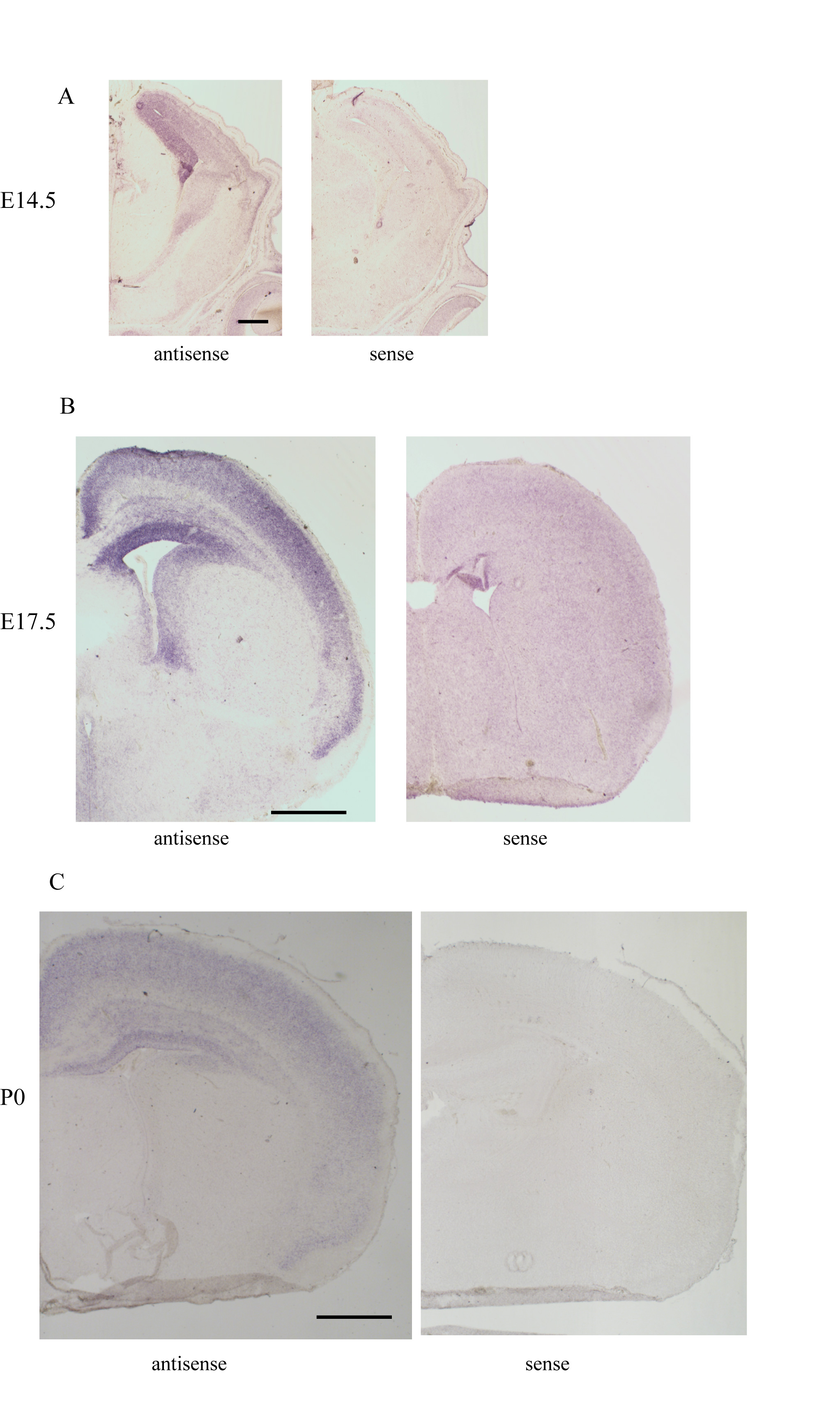

Supplement: FIGURE S1 — (A–C) In situ hybridization shows Tcf4 expression in the developing cerebral cortex of mice. Scale bars, 200 μm. [file Image_1.JPEG]

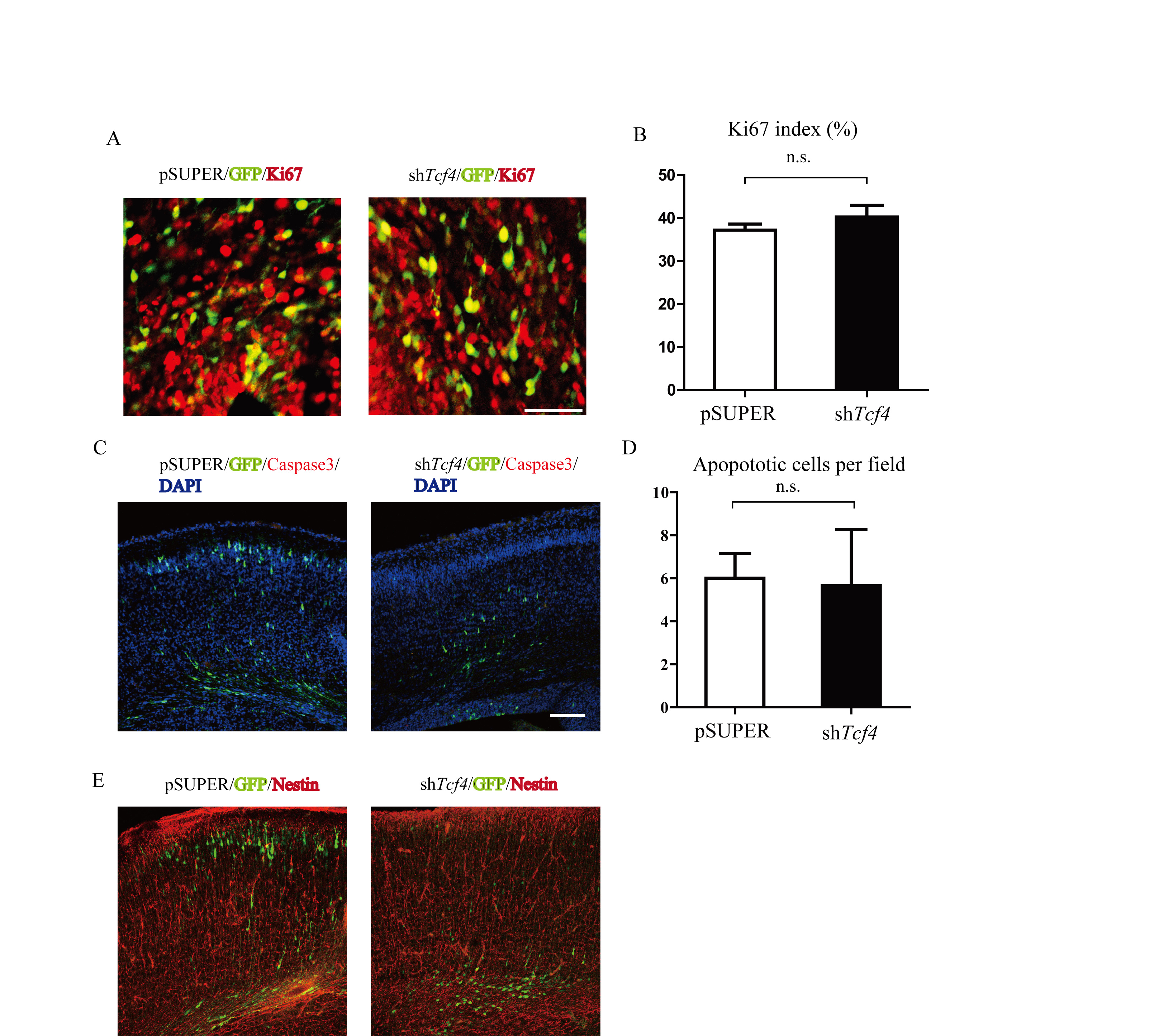

Supplement: FIGURE S2 — (A–E) In utero electroporation at E14.5 with shTcf4 or pSUPER as indicated. The results were quantified by counting the number of stained cells in a constant area of each section, and averaged across sections from at least three different embryos for each antibody. Immunostaining at E17.5 for Nestin, cleaved Caspase 3 or Ki67.Scale bars, 100 μm. Data are shown as the mean ± SEM. n.s., not significant; *p < 0.05, **p < 0.01, and ***p < 0.001; CP, cortical plate; VZ, ventricular zone; IZ, intermediate zone. [file Image_2.JPEG]

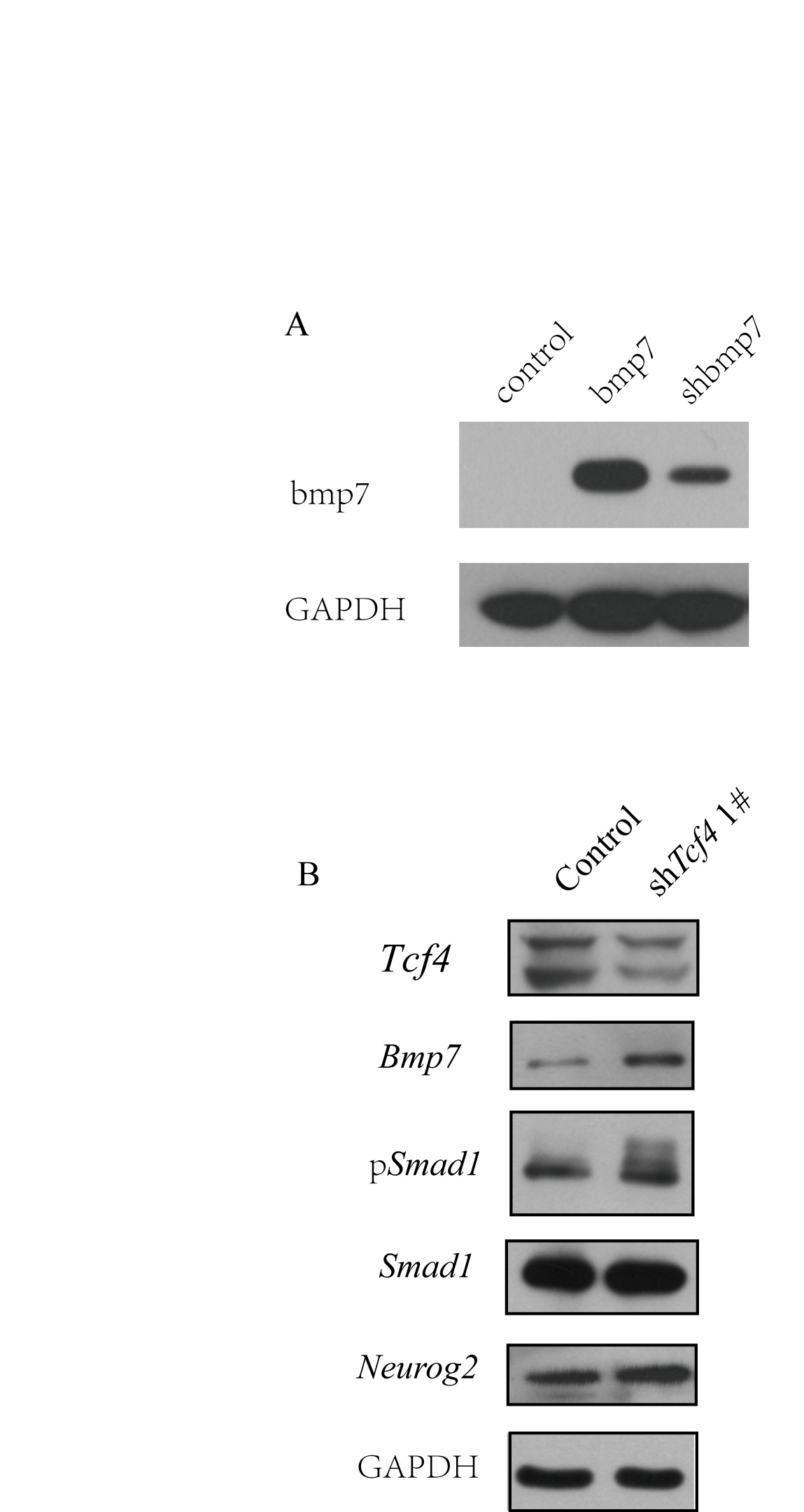

Supplement: FIGURE S3 — (A) Immunoblot analysis in HEK293T cells shows that Bmp7-specific shRNA, shBmp7 is sufficient to knockdown Bmp7. Cells were co-transfected with Bmp7-FLAG and individual shRNA and total cell lysates were prepared for immunoblotting 48 h after transfection. (B) Tcf4 knockdown increased Bmp7 protein levels in primary neuronal progenitor cells. Western blot analysis of the protein extracts from progenitor cells infected with control and Tcf4-shRNA lentivirus. [file Image_3.JPEG]

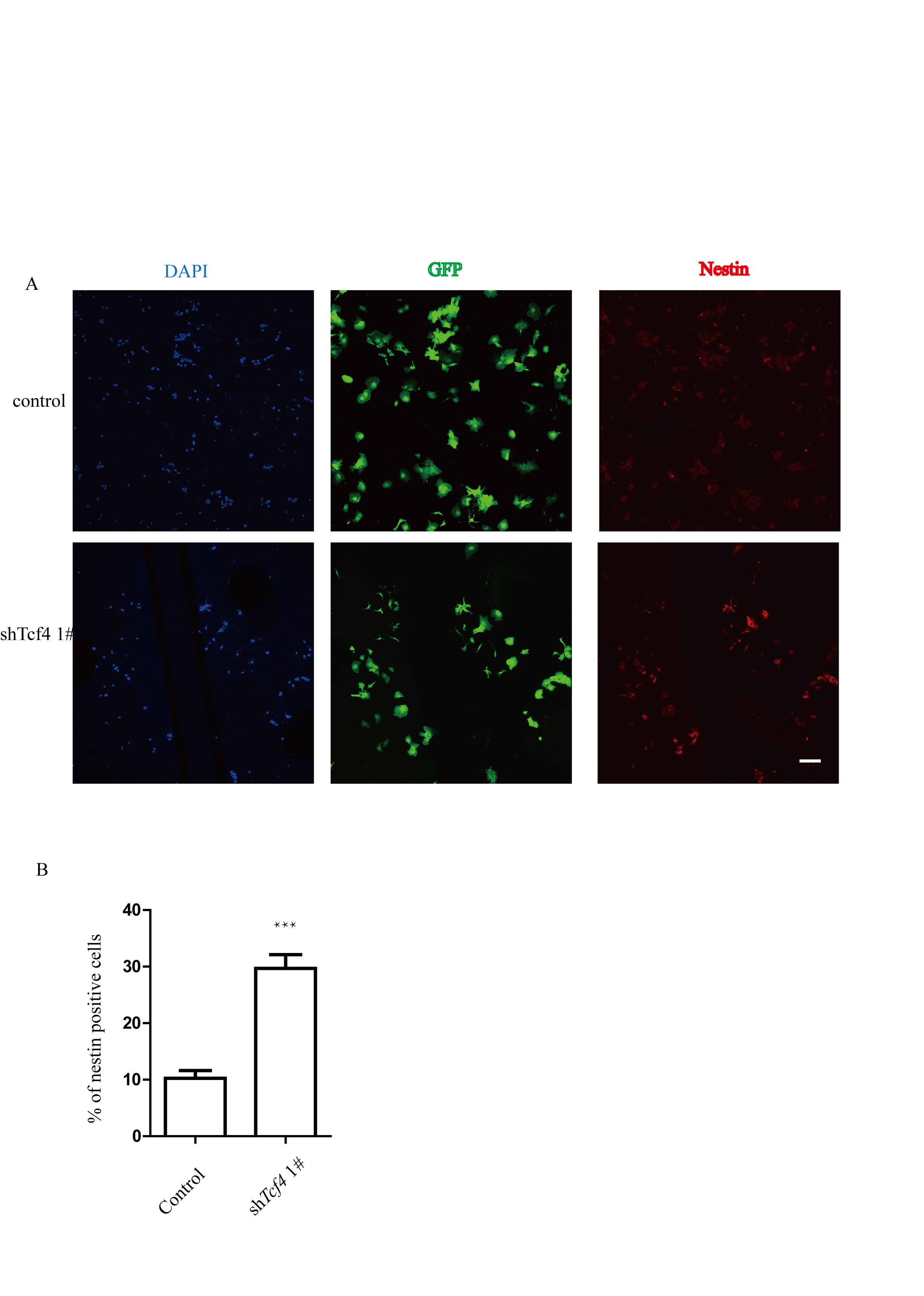

Supplement: FIGURE S4 — (A,B) Immunocytochemistry for Nestin (red) on control and shTcf4 neurosphere cultures after 5 days under differentiation conditions. Scale bars, 100 μm. (B) Quantification of Nestin positive cells in (A). Data are shown as the mean ± SEM. n.s., not significant; *p < 0.05, **p < 0.01, and ***p < 0.001; Student’s t-test or one-way ANOVA, followed by an LSD post hoc test. [file Image_4.JPEG]

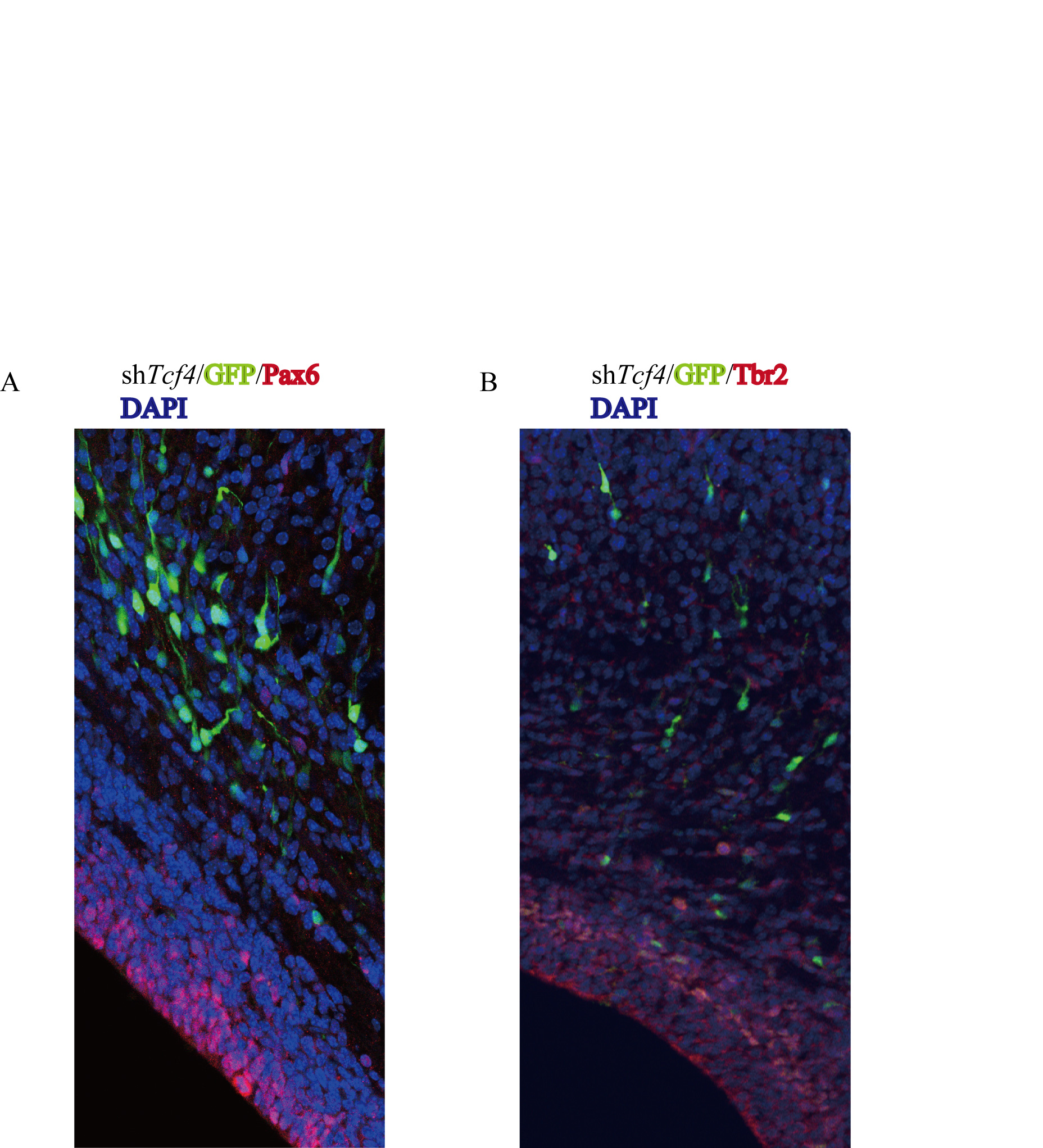

Supplement: FIGURE S5 — (A,B) In utero electroporation at E14.5 with shTcf4 as indicated. Immunostaining at E17.5 for Pax6 or Tbr2 (markers for VZ). VZ, ventricular zone. [file Image_5.JPEG]
